# Accuracy, Quality, and Misinformation of YouTube Abortion Procedural Videos: Cross-Sectional Study

**DOI:** 10.2196/50099

**Published:** 2024-10-22

**Authors:** Nicole Acero, Emma Herrero, Juanita Foncham, Jamie McIlvaine, Emre Kayaalp, Melissa Figueora, Antonia Francis Oladipo

**Affiliations:** 1 Hackensack Meridian School of Medicine Nutley, NJ United States; 2 Department of Family Medicine Boston University School of Medicine Boston Medical Center Boston, MA United States; 3 Syracuse University Syracuse, NY United States; 4 Department of Obstetrics and Gynecology Hackensack University Medical Center Hackensack, NJ United States; 5 Department of Obstetrics and Gynecology Jersey City Medical Center Rutgers Health Jersey City, NJ United States; 6 Department of Obstetrics and Gynecology Overlook Medical Center Atlantic Health System Summit, NJ United States

**Keywords:** abortion, YouTube, social media, accuracy, quality, misinformation, reliability, obstetrics, women's health, reproductive, patient education, health information, prochoice

## Abstract

**Background:**

The internet is often the first source patients turn to for medical information. YouTube is a commonly used internet-based resource for patients seeking to learn about medical procedures, including their risks, benefits, and safety profile. Abortion is a common yet polarizing medical procedure. People interested in obtaining an abortion are likely to use the internet to learn more about abortion procedures and may encounter misinformed and biased information. This is troubling as information found on the internet can significantly alter perceptions and understanding of these procedures. There is no current research that evaluates the accuracy, quality, and misinformation of instructional abortion videos available to patients.

**Objective:**

The purpose of this study was to assess if any given video can deliver accurate and quality information about this topic in an unbiased manner and to assess the level of factually incorrect, distorted, or medically irrelevant information in any given video.

**Methods:**

Procedural methods of abortion were queried on YouTube on August 22, 2022. The videos were screened with strict exclusion criteria. Videos were categorized into “video slants” based on the language and attitudes expressed in each video. Video accuracy was calculated using the Surgical Curriculum in Obstetrics and Gynecology (SCOG) checklist for each corresponding procedure. Video quality was calculated using the Laparoscopic Surgery Video Educational Guidelines (LAP-VEGaS) criteria. The level of misinformation was assessed with the evidence-based Anti-Choice Rubric, which scores the amount of factually incorrect, distorted, or medically irrelevant information in each video.

**Results:**

A total of 32 videos were analyzed and categorized into 3 “video slant” groups: neutral (n=23, 72%), antichoice (n=4, 12%), and prochoice (n=5, 16%). Using the SCOG checklist, neutral videos had the highest median accuracy (45.9%), followed by antichoice videos (24.6%) and prochoice videos (18.5%). None of the videos met the LAP-VEGaS quality control criteria, (score>11, indicating adequate quality). Neutral videos had a median score of 8.8 out of 18, with antichoice videos scoring 10.75 and prochoice videos scoring 6.2. Using the Anti-Choice Rubric, neutral videos mentioned only 1 factually incorrect piece of information. Antichoice videos mentioned 12 factually incorrect pieces of information, 8 distortions, and 3 medically irrelevant pieces of information. Prochoice videos did not mention any of the 3 themes.

**Conclusions:**

Using the SCOG checklist, the accuracy of instructional videos were inconsistent across the 3 identified “video slants.” Using LAP-VEGaS criteria, the quality of educational videos were also inconsistent across the 3 “video slants.” Prochoice videos had the lowest level of misinformation, with no mentions of any of the 3 themes. Antichoice videos had the highest levels of misinformation, with mentions in all 3 themes. Health care professionals should consider this when counseling patients who may watch YouTube videos for information regarding abortion procedures.

## Introduction

The internet is often the first source patients turn to for medical information. One in 3 American adults have used the internet to better understand a medical condition, with nearly half seeking information about specific procedures [1]. Abortion is one of the most common medical procedures globally [2]. People interested in obtaining an abortion are likely to use the internet to learn more about abortion procedures, including their safety and complications [3]. Internet searches on abortion-related terms spiked following the leaked Supreme Court opinion in Dobbs v. Jackson Women’s Health Organization in May 2022 [4].

Procedural abortion includes manual vacuum aspiration, electric vacuum aspiration (colloquially referred to as dilation and curettage [“D&C”]), and dilation and evacuation (“D&E”). Scientific evidence demonstrates that induced abortion is safe. While the risks of abortion increase with gestational age, abortion at any gestational age is safer than childbirth [5]. Many antichoice arguments rest on misinformation regarding the safety and health consequences of abortion. Additionally, false or misleading information on the internet about abortion exerts an influence on public debates and policies [6].

Abortion knowledge in the United States is low overall. There is a large gap between the scientific evidence of abortion safety and public perception, and political beliefs are not significantly associated with abortion knowledge [7]. People seeking information about the safety and potential risks of abortion are likely to encounter a substantial volume of misinformed and biased information. This is troubling as information found on the internet can significantly alter perceptions and understanding of procedures [3].

YouTube is commonly used as a freely accessible internet-based resource for patients seeking information about medical procedures, including their risks, benefits, and safety profiles. One study found that 7% of participants surveyed used YouTube to aid their search on abortion safety [3]. There is no current research that evaluates the accuracy, quality, or level of misinformation of instructional videos available to patients. Given the reliance of information gathered on the internet to inform medical decision-making, this study was designed to assess whether any particular video on YouTube can deliver accurate, quality, and factually correct information about this topic in an unbiased manner.

## Methods

### YouTube Search

Surgical methods of abortion were used as a search query on YouTube in incognito mode, so the searcher’s own algorithm would not affect the results. Search terms included “surgical abortion,” “video demonstration + manual vacuum aspiration/D&C/D&E,” “how to perform abortion manual vacuum aspiration OR D&C,” “how to perform a D&E,” and “manual vacuum aspiration/D&C/D&E + surgery.” Videos were excluded if they were not in English, duplicate of another video, noninstructional in nature, or unrelated in topic.

### Ethical Considerations

The creators of the videos analyzed in this study have been deidentified. No ethics approval was applied for, as this study did not involve human subjects.

### Video Categorization

Videos were categorized into 3 “video slant” groups: neutral, antichoice, and prochoice, based on the methodology by Han et al [2]. Videos were determined to have a prochoice or antichoice bias if either (1) information was given in a biased or dramatic fashion or (2) the video displayed an opinion regarding the provision of abortion and its legality. Otherwise, the video was considered neutral. We assigned a slant clarity rating based on how easy it was to discern the video slant: (1) obvious, (2) in between, or (3) difficult to tell.

### Data Analysis

Each video’s accuracy was calculated based on how many steps on the corresponding Surgical Curriculum in Obstetrics and Gynecology (SCOG) checklist were included in each video (Multimedia Appendix 1). Each video’s quality was calculated using the LAP-VEGaS criteria (Multimedia Appendix 2) [8]. There is significant correlation between a score of ≥11 out of 18 on the LAP-VEGaS tool and recommended acceptance for publication, indicating adequate quality of educational content. The Anti-Choice Rubric was created to grade the level of misinformation within each video. The rubric was created based on talking points gathered from antichoice organizations and categorized into 3 themes: factually incorrect, distortion, and medically irrelevant information (Multimedia Appendix 3). Videos were scored for the number of times a given viewpoint was mentioned.

## Results

### Video Characteristics

After removing duplicates, our search yielded 37 videos. Of those, 32 videos met inclusion criteria and were analyzed (Table 1). Videos were categorized into 3 “video slant” groups: neutral (n=23, 72%), antichoice (n=4, 12%), and prochoice (n=5, 16%). Clarity was obvious (n=26, 81%), difficult to tell (n=4, 12%), or in between (n=2, 6%). Of the 4 videos deemed “difficult to tell,” 3 (75%) had a prochoice slant. The 2 videos deemed “in between” were neutral.

**Table 1:** YouTube video characteristics.

Characteristics		All videos (n=32)	Neutral videos (n=23)	Antichoice videos (n=4)	Prochoice videos (n=5)
**Creator type, n (%)**					
	Physician	21 (66)	15 (65)	3 (75)	4 (80)
	Nonphysician	5 (16)	4 (17)	1 (25)	1 (20)
	Unspecified	6 (19)	4 (17)	0 (0)	0 (0)
**Video type, n (%)**					
	Live	7 (22)	7 (30)	0 (0)	0 (0)
	Digital reconstruction	5 (16)	3 (13)	2 (50)	0 (0)
	Model or simulation	3 (9)	2 (9)	1 (25)	0 (0)
	Combined	4 (13)	4 (17)	0 (0)	0 (0)
	Lecture with images	12 (38)	7 (30)	1 (25)	4 (80)
	Lecture without images	1 (3)	0 (0)	0 (0)	1 (20)
**Channel type, n (%)**					
	Education	10 (31)	7 (30)	0 (0)	3 (60)
	Physician	8 (25)	7 (30)	0 (0)	1 (20)
	Personal	7 (22)	6 (26)	1 (25)	0 (0)
	Clinic	1 (3)	1 (4)	0 (0)	0 (0)
	Medical	1 (3)	1 (4)	0 (0)	0 (0)
	Other	5 (16)	1 (4)	3 (75)	1 (20)
**Country, n (%)**					
	United States	8 (25)	4 (17)	3 (75)	1 (20)
	England	2 (6)	1 (4)	0 (0)	1 (20)
	Pakistan	2 (6)	2 (9)	0 (0)	0 (0)
	India	3 (9)	2 (9)	0 (0)	1 (20)
	New Zealand	1 (3)	0 (0)	1 (25)	0 (0)
	Moldova	1 (3)	0 (0)	0 (0)	1 (20)
	Ethiopia	1 (3)	1 (4)	0 (0)	0 (0)
	Kenya	1 (3)	1 (4)	0 (0)	0 (0)
	Unspecified	13 (41)	12 (52)	0 (0)	1 (20)
**Views, median (IQR)**		1,476,321 (3689.5-5,061,341)	94,465 (22,108-608,745)	977,863.5 (56,619-5,111,341)	526 (207-2928)
**Likes, median (IQR)**		270.5 (22-1100)	492 (165-460)	373.5 (29.5-5844)	7 (0-55,008.5)

### Evaluation Outcomes

Using the SCOG checklist, neutral videos had the highest median accuracy (45.9%), followed by antichoice videos (24.6%) and prochoice videos (18.5%).

Of the 32 videos, none met the LAP-VEGaS quality control criteria. Neutral videos had a median score of 8.8 out of 18, with antichoice videos scoring 10.75 and prochoice videos scoring 6.2.

Using the Anti-Choice Rubric, neutral videos mentioned 1 factually incorrect piece of information. Antichoice videos mentioned 12 factually incorrect pieces of information, 8 distortions, and 3 medically irrelevant pieces of information. Prochoice videos did not mention any of the 3 themes.

## Discussion

### Principal Findings

Our study of YouTube videos providing information about procedural abortions found that a majority (23/32, 72%) were neutral. Based on the SCOG checklist, neutral videos were the most accurate, followed by antichoice videos. Prochoice videos were the least accurate. Based on the LAP-VEGaS criteria, antichoice videos had the highest quality, followed by neutral videos. Prochoice videos had the lowest quality. While prochoice videos had the lowest accuracy and quality, they contained no misinformation according to our Anti-Choice Rubric. Antichoice videos contained the highest level of misinformation. The neutral videos had 1 mention of factually incorrect information.

Based on these findings, we can conclude that neutral videos are more reliable than either antichoice or prochoice videos. Antichoice videos may be of higher quality and more accurately reflect a procedure compared to prochoice videos. However, they also contained the largest amount of misinformation. Prochoice videos, in contrast, omitted more steps involved in a given procedure but contained no misinformation. Therefore, accuracy and quality alone cannot be used to determine whether any given video is trustworthy.

One limitation of our analysis was the small sample size, which limited the ability to perform association analysis. Additionally, this study offers a snapshot of the videos available at the time of our analysis. Furthermore, most of our researchers are involved in abortion-related research. We recognize that nonexperts may have rated and classified these videos differently.

The strengths of this study include the use of validated tools, namely, the SCOG checklist and the LAP-VEGaS criteria, to measure accuracy and quality, respectively.

### Comparison to Prior Work

To our knowledge, there is no published research evaluating the trustworthiness of individual YouTube procedural abortion videos for their informational content. Quality analyses of YouTube videos have been conducted in urology and obstetrics/gynecology, although there are no known studies on abortion procedures [9,10]. A similar work by Han et al [2] sought to describe the trustworthiness of web-based information on abortion safety and risks of depression and infertility following abortion. This study determined trustworthiness by factors such as content and source of content. Our study sought to determine the trustworthiness of any given video based on accuracy, quality, and level of misinformation. Accuracy and quality were determined using 2 validated tools (the SCOG checklist and LAP-VEGaS criteria, respectively). The level of misinformation was determined by an evidence-based rubric. This study shows that accuracy, creator type, references, or affiliation with health care organizations or health care professionals alone cannot determine whether any given video is trustworthy.

### Conclusions

Our results show that the accuracy or quality of a video alone are not reliable factors in determining trustworthiness. Videos that appear trustworthy based on these factors alone may contain language or imagery that provides misinformation or lends itself to misinterpretation, despite also containing accurate technical information. Furthermore, a given video’s slant may provide motivation to either downplay elements of a procedure to not scare a patient away from a procedure or emphasize the details of the procedure for the opposite reason.

A shared decision-making model combines provider clinical expertise with patient values. It is the job of clinical providers to educate their patients, and it is the patients’ responsibility to use that information in combination with their values to make an informed decision about their health. Therefore, it is important for providers to be aware of the type of information patients may encounter when making decisions to address patient concerns. It is also important for providers to be able to direct patients toward accurate and quality information to assist patients in their own self-education processes. In the future, we hope to use the information from these findings to develop a tool for patients and physicians to grade the trustworthiness of informational content in real time. We hope the use of this tool will encourage content creators to develop content that is not just impactful but—more importantly—truthful.

## Appendix

Multimedia Appendix 1 The Surgical Curriculum in Obstetrics and Gynecology (SCOG) checklist for procedural abortions, which was used to determine the accuracy of a YouTube video.

Multimedia Appendix 2 The Laparoscopic Surgery Video Educational Guidelines (LAP-VEGaS) criteria, which were used to determine the quality of a video.

Multimedia Appendix 3 The evidence-based Anti-Choice Rubric, which was used to determine the level of abortion misinformation in a given video.

Multimedia Appendix 4 Dataset of YouTube procedural abortion videos included in this cross-sectional study.

## Abbreviations

LAP-VEGaS: Laparoscopic Surgery Video Educational Guidelines
SCOG: Surgical Curriculum in Obstetrics and Gynecology

## Data Availability

The dataset used in this study is available in Multimedia Appendix 4.
