# Supplementary material for: Accuracy, Quality, and Misinformation of YouTube Abortion Procedural Videos: Cross-Sectional Study
Source: J Med Internet Res. 2024 Oct 22;26:e50099. doi: 10.2196/50099 (PMC11538871; doi:10.2196/50099)
Supplement: Multimedia Appendix 1 [file jmir_v26i1e50099_app1.pdf]

First Trimester Uterine Aspiration

1	Assemble all materials
2	Conduct “timeout”
3	Bimanual exam for size/position of uterus
4	Insert speculum and prep cervix with iodine or alternative in case of allergy
5	Place tenaculum
	Inject 2ml lidocaine at planned tenaculum site
	Good purchase to anterior or posterior lip
	Close ratchets slowly to lessen pain
	Exert gentle traction on cervix for dilation and manipulate cervix to administer paracervical block
6	Perform Paracervical block
7	Dilate the cervix
	Hold dilator loosely using tapered dilator
	Redirect dilator if significant resistance
8	Prepare MVA syringe
	Attach cannula
	Close (compress) valve
	Pull back plunger
	Attach appropriate tubing
	Test suction: Adjust to 40-60 mm Hg
9	Select Syringe (Typically gestational age (GA) or GA + 1 mm)
10	Inserts cannula, performs curettage
11	Rotation combined with gentle in and out motion
	Ultrasound may be used
	Rotate until signs the uterus is empty
12	Complete procedure
	Remove tenaculum
	Apply hemostatic treatment if needed
	Assess for uterine bleeding

	Remove speculum
13	Examine tissue to ensure gestational sac is present
	Strain and rinse the tissue
	Place tissue in a clear container
	It is recommended to use a backlight to inspect the tissue

Second Trimester Dilation and Evacuation

1	Begin counseling the patient on all of the risks, benefits, and alternative to procedure
2	Ensure that all equipment has been set up
3	Perform bimanual exam
4	Conduct a timeout
5	Prep vagina and cervix for sterile technique
6	Insert speculum
7	Use single tooth tenaculum to grab anterior lip of cervix
8	Starting with smallest dilator, serially dilate cervical os, being sure not to perforarate through the uterus
9	Curettage - smooth or sharp curette and passing it through the os
	Advance the curette to the funds then withdraw back, scraping the inner lining
	Repeat this motion within each quadrant of the uterus until the entire cavity has been curetted
	Repeat passes until satisfied with tissue amount and lining has a gritty texture
	Inspect currettings to ensure there is enough tissue
10	Empty tissue into formalin container
11	Take tenaculum off cervix, inspect for hemostasis, remove speculum

Second Trimester Dilation and Evacuation

1	Bimanual examination for size and position of uterus and assess cervical dilation and effacement
2	Assess location of fetus using ultrasound examination or digital examination
3	Grasp anterior lip of cervix with appropriate instrument (speculum and grasper)
4	Administer lidocaine with vasopressin paracervically
5	Pass tapered dilators if additional dilation needed
6	Attempt to perform amniotomy
7	Choose proper instrument for extraction
	Initiate extraction by introducing instrument and putting traction on anterior lip Introduce forceps closed
8	Open widely when past internal cervical os
9	Extract material carefully using appropriate technique
	Keep track of parts removed
10	Confirm the uterus is empty
11	Examine tissue removed to ensure all parts have been extracted
12	Remove all instruments
