# Supplementary figures and images for: Accuracy, Quality, and Misinformation of YouTube Abortion Procedural Videos: Cross-Sectional Study
Source: J Med Internet Res. 2024 Oct 22;26:e50099. doi: 10.2196/50099 (PMC11538871; doi:10.2196/50099)

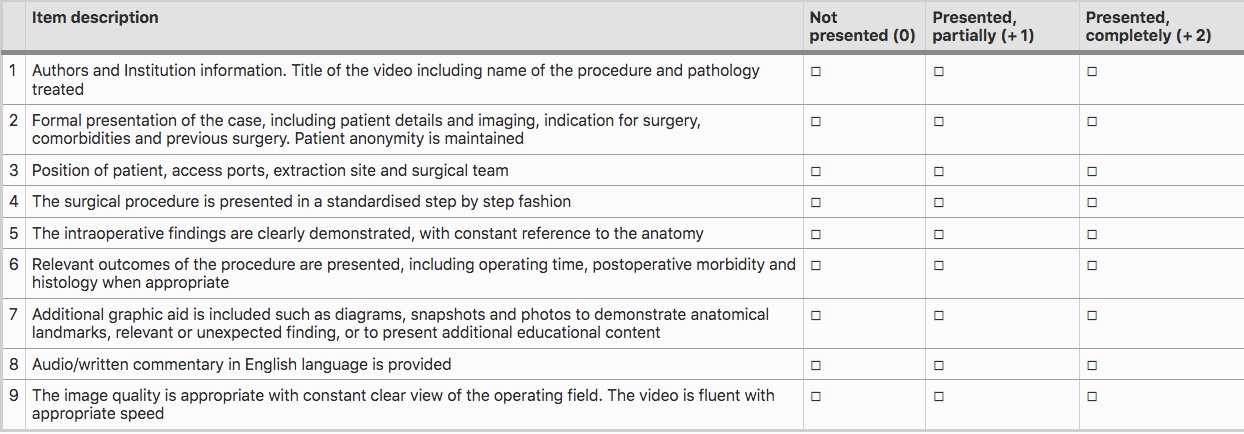

Supplement: Multimedia Appendix 2 [file jmir_v26i1e50099_app2.png]
