# Supplementary material for: Accuracy, Quality, and Misinformation of YouTube Abortion Procedural Videos: Cross-Sectional Study
Source: J Med Internet Res. 2024 Oct 22;26:e50099. doi: 10.2196/50099 (PMC11538871; doi:10.2196/50099)
Supplement: Multimedia Appendix 3 [file jmir_v26i1e50099_app3.pdf]

Factually Incorrect	Distortion	Medically Irrelevant
1. Abortion takes a permanent physical toll on women's bodies.<br>2. Fetal pain begins as early as 12 weeks.<br>3. Obstetricians and Gynecologists worldwide agree that it is never medically necessary to intentionally destroy an unborn child to save a mother's life.<br>4. Women who have abortions are more likely to experience subsequent mental health problems.<br>5. All women, especially young teenagers, are at risk for damage to their cervix during an abortion, which can lead to complications with later pregnancies.	1. Every person should be protected/respected under law, including the innocent unborn child in the womb.<br>2. Abortion disproportionately affects Hispanic and Black women.	1. A baby, from the moment of conception, is self-directed. He/she is not serving the mother's body like an organ, and the mother's brain is not directing the growth.
